# AI assisted surgical guides for dental implant placement

**DOI:** 10.6026/973206300211002

**Published:** 2025-05-31

**Authors:** Saurabh Gupta, Dinesh Yadav, Anubhuti Anubhuti, Lubna Tabassum Siddiqui, Deepa Rudrappa, Abdul KalamAzad, Alkananda Sahoo

**Affiliations:** 1 Consultant, Periodontist and Implantologist, Jammu, Jammu and Kashmir, India; 2 Department of Oral and Maxillofacial Surgery, Ekalavya Dental College & Hospital, Rajasthan, India; 3 Consultant, Prosthodontist, Dental Smile, Chandigarh, Haryana, India; 4 Department of Periodontics, Swargiya Dadasaheb Kalmegh Smruti Dental College and Hospital, Wanadongri, Hingna, Nagpur, Maharashtra, India; 5 Department of Periodontics and Implantology, SJM Dental College and Hospital, Chitradurga-577501, Karnataka, India; 6 Department of Oral and Maxillofacial Surgery, College of Dentistry, Qassim University, Saudi Arabia; 7 Department of Oral and Maxillofacial Pathology, Kalinga Institute of Dental Sciences, KIIT Deemed to be University, Patia, Bhubaneswar-751024, Odisha, India

**Keywords:** AI-assisted surgical guides, dental implants, implant stability, accuracy, resonance frequency analysis, insertion torque, depth deviation, angular deviation, implant success rate

## Abstract

Dental implant placement requires high precision and advancements in technology, such as AI-assisted surgical guides. Therefore, it is of interest to compare AI-assisted surgical guides, conventional guides and freehand techniques in dental implant placement. Hence, 75 artificial jaw models were divided into three groups of 25 each. The implants were placed using standardized methods. The AI guides were created using machine learning software (PlanmecaRomexis®, India). Statistical analysis (SPSS v25) used Shapiro-Wilk, Levene's test, ANOVA with Tukey, p<0.05, and ICC for reliability.AI-assisted surgical guides offer superior accuracy, stability and implant success rates.

## Background:

Dental implantology is a specialized area that demands a high level of precision and accuracy to ensure that dental implants are successful and functional over the long haul [1]. The success of placing dental implants hinges not just on the clinician's skill but also on the tools and techniques they use during the procedure. Traditionally, methods like freehand placement and manually made surgical guides have been the go-to for accurately positioning dental implants [2]. However, these older techniques often come with a higher chance of variability and human error, especially when it comes to critical factors like the angle, depth and overall placement of the implant. This can lead to complications such as improper implant positioning, poor osseointegration and longer recovery times for patients [3]. To tackle these challenges and technology has brought forth innovative solutions that aim to enhance the precision and reliability of dental implant procedures. AI-assisted surgical guides leverage machine learning and 3D imaging for precise implant planning. This advancement enhances accuracy and positioning in dental procedures [3, 4- 5]. AI-generated guides are designed through detailed analysis of the patient's anatomy. They ensure a personalized and precise approach to implant placement. By automating the planning process, these guides help reduce human error and provide precise, data-driven recommendations for where to place the implants [6, 7]. The exciting potential of AI-assisted implantology lies in its ability to deliver greater accuracy in implant placement compared to traditional methods [8]. AI-assisted guides are believed to enhance implant stability, lower the risk of complications and ultimately boost the overall success rates of dental implants. As AI technology continues to make waves in the healthcare and dental fields, its integration into dental implantology marks a significant advancement [7, 8]. Despite AI's growing role in dentistry, evidence-based studies on its effectiveness in surgical guides remain limited. More research is needed to compare AI-assisted guides with traditional methods and assess their clinical benefits [8]. Therefore, it is of interest to compare AI-driven guides with conventional techniques to fill gaps in the literature. It explores AI's potential to enhance dental implant procedures.

## Materials and Methods:

This *in-vitro* study was conducted under controlled lab conditions. Seventy five artificial jaw models were divided into three groups: AI-assisted (n=25), conventional guides (n=25) and freehand placement (n=25). The goal was to evaluate how accurately and stably implants could be placed using these different techniques. Before the procedures, we assessed the artificial jaw models to understand their baseline anatomical structures and then we performed the implant placements using standardized methods. The AI-assisted surgical guides were crafted with the help of machine learning-based planning software (PlanmecaRomexis®, India), which allowed for precise implant planning. On the other hand, the conventional surgical guides were made using traditional CAD/CAM technology (3Shape®, India). For the freehand placement group, experienced implantologists placed the implants without any surgical guides. All implants used in this study were titanium-based Nobel Biocare® (India) implants, ensuring consistency across all groups. The guides were 3D-printed using biocompatible photopolymer resin (NextDent SG®, India), providing a reliable and stable medium for the implant placements. To e valuate implant stability, we measured several parameters. We used Resonance Frequency Analysis (RFA) with the Osstell ISQ device to assess stability and obtain Implant Stability Quotient (ISQ) values. During the implant placement, we recorded Insertion Torque Values (ITV), which gave us insights into the primary stability achieved during insertion. For evaluation of accuracy; we measured angular deviation by comparing the planned implant angle with the actual angle after placement using post-placement imaging. Similarly, we calculated depth deviation by comparing the intended depth with the actual depth of the implant placement. These measurements were taken using calibrated digital calipers and advanced imaging techniques to ensure precision. The implant success rate was defined by stable osseointegration; angular deviation and depth deviation. All statistical analysis was performed using SPSS (IBM SPSS v25, India). Data normality was assessed using the Shapiro-Wilk test, while homogeneity of variance was verified with Levene's test. Comparisons between groups were made using one-way ANOVA with post-hoc Tukey tests to determine significant differences. A p-value of <0.05 was considered statistically significant. To ensure reliability, inter-observer measurements were assessed using the intra-class correlation coefficient (ICC).

## Results:

Group A showed the highest Resonance Frequency Analysis (RFA) values, averaging 78 ± 5 ISQ. This indicated superior stability over conventional guides (72 ± 6 ISQ) and freehand placement (65 ± 8 ISQ). Additionally, the insertion torque values (ITV) were also highest in Group A at 45 ± 3 Ncm, suggesting better primary stability during the insertion process. Group A demonstrated the highest accuracy with the smallest angular deviation of 2.1 ± 0.8°. Conventional guides measured 3.4 ± 1.2°, while freehand placement showed the largest deviation at 6.5 ± 2.4°. Group A exhibited the least depth deviation at 0.3 ± 0.1 mm, followed by conventional guides at 0.6 ± 0.2 mm. Freehand placement had the highest deviation at 1.1 ± 0.4 mm, highlighting AI-assisted guides' superior control. Group A achieved the highest implant success rate at 96%, followed by conventional guides at 90%. Freehand placement had the lowest success rate at 80%, highlighting AI-assisted guidance's superior stability and precision (Table 1) (Figure 1-5 see PDF). One-way ANOVA with post-hoc Tukey tests was used to compare AI-assisted guides, conventional guides, and freehand placement. Significant differences were found across all measured parameters (p < 0.05). RFA ISQ values, insertion torque (ITV), angular and depth deviations, and success rates showed significant differences. Additionally, inter-observer reliability was assessed using the intra-class correlation coefficient (ICC), with values greater than 0.90, demonstrating high consistency in the measurement process. The post-hoc Tukey test showed significant differences (p < 0.05) across all parameters, confirming the superior performance of AI-assisted guides.AI-assisted guides had the greatest advantage over freehand placement, demonstrating significantly better stability, accuracy, and success rates. Conventional guides performed better than freehand placement but remained inferior to AI-assisted guides in all measured outcomes (Table 2).

## Discussion:

Dental implant success relies on precise placement, stability and osseointegration. AI-assisted surgical guides have emerged as a superior alternative to conventional guides and freehand placement, offering enhanced accuracy and predictability [1, 8]. In this study, AI-assisted guides (Group A) demonstrated the highest implant stability with an ISQ of 78 ± 5, outperforming conventional guides (72 ± 6) and freehand placement (65 ± 8). AI has greatly enhanced the precision of image segmentation, playing a key role in increasing the objectivity and automation of surgical procedures.

Medical images are more complex than natural images, making segmentation challenging. Traditional methods like thresholding, region growing, and max-flow offer limited accuracy [9]. The insertion torque values (ITV) were also highest in Group A (45 ± 3 Ncm), indicating an enhanced primary stability with improved bone engagement and anchorage. AI-driven treatment planning for dental implant placement shows significant potential in improving accuracy, efficiency and accessibility [10]. Regarding accuracy, AI-assisted guides minimized angular deviation (2.1 ± 0.8°); significantly lower than conventional guides (3.4 ± 1.2°) and freehand placement (6.5 ± 2.4°). This result aligns with Satapathy *et al.* (2024) findings, which demonstrated that AI-guided placement reduces deviation potentially reducing the margin of error associated with manual planning [10]. Similarly, depth deviation was least in Group A (0.3 ± 0.1 mm) compared to 0.6 ± 0.2 mm (conventional) and 1.1 ± 0.4 mm (freehand). AI-powered guides combined with computer-assisted implant surgery (CAIS) improve implant placement precision. They minimize deviations in position, angle, and depth compared to conventional techniques [11]. The highest implant success rate (96%) in Group A compared to 90% (conventional) and 80% (freehand) emphasizing the long-term benefits of AI precision. Artificial intelligence (AI) is transforming healthcare by enhancing productivity and offering innovative methods for service delivery. In dentistry, AI is revolutionizing early diagnosis and predicting dental implant needs. Research by Alharbi *et al.* (2022) led to development of four machine-learning algorithms -Bayesian network, random forest, AdaBoost and improved AdaBoost-to predict when patients may require dental implants. The results demonstrate that the improved AdaBoost algorithm outperforms the others, achieving an accuracy of 91.7%. This work aims to assist healthcare managers and decision-makers in identifying patients requiring implants based on specific diagnoses [12]. AI has advanced dentistry by aiding in periodontal disease detection, classification, and risk assessment. It also helps evaluate bone levels, detect halitosis, and plan dental implant treatments. AI optimizes implant designs, identifies implant types, and predicts treatment outcomes [13]. Macrì *et al.* (2024), stated integration of artificial intelligence in implant planning holds great potential for enhancing clinical results and streamlining patient care [14]. Additionally, AI plays a crucial role in the efficient management of dental practices. These developments highlight the potential to enhance the use of AI-assisted surgical guides in dental implant surgery, improving accuracy, precision and overall outcomes. The AI-assisted guides demonstrated superior accuracy and success rates compared to other techniques. The reduced angular and depth deviations indicate improved precision, which may enhance long-term implant stability. Future studies should investigate AI integration with real-time robotic assistance for further refinement. While the study provides valuable insights, one limitation is that the study was conducted using artificial jaw models, which may not fully replicate the complexities and variations found in a clinical setting. Factors such as bone type; jawbone depth and patient-specific anatomy could potentially affect the outcomes of implant placement in real-world scenarios. The introduction of AI-assisted surgical guides is a game changer in the world of dental implantology, paving the way for greater precision and reliability. As implant dentistry keeps advancing, the integration of AI is set to establish new benchmarks for efficiency, safety and predictability, ushering in a future where technology and clinical expertise come together to transform patient care.

## Conclusion:

We discussed the advantages of using AI technology compared to traditional methods of implant placement, especially when it comes to improving crucial factors like implant stability, positioning accuracy and overall success rates. Data shows significant enhancements when using AI-driven techniques, indicating that these advanced tools not only reduce the risks associated with human error but also enhance patient outcomes.

## Figures and Tables

**Table 1:** Implant stability and accuracy measurements

**Parameter**	**AI-assisted Guides (Group A)**	**Conventional Guides (Group B)**	**Freehand Placement (Group C)**
Resonance Frequency Analysis (RFA) (ISQ)	78±5 ISQ	72±6 ISQ	65±8 ISQ
Insertion Torque Values (ITV) (Ncm)	45±3 Ncm	40±4 Ncm	35±5 Ncm
Angular Deviation (°)	2.1±0.8°	3.4±1.2°	6.5±2.4°
Depth Deviation (mm)	0.3±0.1 mm	0.6±0.2 mm	1.1±0.4 mm
Implant Success Rate (%)	96%	90%	80%

**Table 2:** Post-Hoc tukey test results for pairwise comparisons

**Parameter**	**Comparison (Groups)**	**Mean Difference**	**p-Value**	**Significance**
Resonance Frequency Analysis (RFA ISQ)	AI vs. Conventional	6	0.012	Significant
	AI vs. Freehand	13	0.001	Significant
	Conventional vs. Freehand	7	0.018	Significant
Insertion Torque Values (ITV)	AI vs. Conventional	5	0.015	Significant
	AI vs. Freehand	10	0.002	Significant
	Conventional vs. Freehand	5	0.025	Significant
Angular Deviation (°)	AI vs. Conventional	-1.3	0.021	Significant
	AI vs. Freehand	-4.4	0.001	Significant
	Conventional vs. Freehand	-3.1	0.008	Significant
Depth Deviation (mm)	AI vs. Conventional	-0.3	0.017	Significant
	AI vs. Freehand	-0.8	0.001	Significant
	Conventional vs. Freehand	-0.5	0.022	Significant
Implant Success Rate (%)	AI vs. Conventional	6	0.011	Significant
	AI vs. Freehand	16	0.001	Significant
	Conventional vs. Freehand	10	0.005	Significant
